# Exploring the potential of fully automated LUMIPULSE G plasma assays for detecting Alzheimer’s disease pathology

**DOI:** 10.1186/s13195-024-01397-9

**Published:** 2024-03-07

**Authors:** Anuschka Silva-Spínola, Maria João Leitão, Alicia Nadal, Nathalie Le Bastard, Isabel Santana, Inês Baldeiras

**Affiliations:** 1 Faculty of Medicine, University of Coimbra, Coimbra, Portugal; 2 Center for Innovative Biomedicine and Biotechnology, University of Coimbra, Coimbra, Portugal; 3 Centre for Informatics and Systems, Department of Informatics Engineering, University of Coimbra, Coimbra, Portugal; 4 Neurochemistry Laboratory, Neurology Department, Coimbra University Hospital, Praceta Mota Pinto, 3004-561 Coimbra, Portugal; 5 Fujirebio Iberia, S.L., Barcelona, Spain; 6 Fujirebio Europe N.V., Ghent, Belgium; 7 Neurology Department, Centro Hospitalar E Universitário de Coimbra, Coimbra, Portugal

**Keywords:** Early detection, Plasma biomarkers, Alzheimer’s disease, Automated

## Abstract

**Background:**

LUMIPULSE G-automated immunoassays represent a widely used method for the quantification of Alzheimer’s disease (AD) biomarkers in the cerebrospinal fluid (CSF). Less invasive blood-based markers confer a promising tool for AD diagnosis at prodromal stages (mild cognitive impairment (MCI)). Highly sensitive assays for the quantification of amyloid-beta (Aβ) and phosphorylated Tau-181 (p-Tau181) in the blood are showing promising results. In this study, we evaluated the clinical performance of the recently available fully automated LUMIPULSE plasma marker assays for detecting brain AD pathology and for predicting progression from MCI to AD dementia stage.

**Methods:**

A retrospective exploratory cohort of 138 individuals (22 neurological controls [NC], 72 MCI, and 44 AD dementia patients) was included. Data regarding baseline CSF concentrations of Aβ42, Aβ40, t-Tau, and p-Tau181 was available and used to establish the presence of AD brain pathology. Baseline Aβ42, Aβ40, and p-Tau181 concentrations were determined in stored plasma samples using high-throughput fully automated LUMIPULSE assays. Progression from MCI to AD dementia was evaluated during follow-up (mean 6.4 ± 2.5 years). Moreover, a prospective validation cohort of 72 individuals with memory complaints underwent AD biomarker quantification, closely mirroring typical clinical practice. This cohort aimed to confirm the study’s main findings.

**Results:**

In the exploratory cohort, correlations between CSF and plasma were moderate for p-Tau181 (*ρ* = 0.61, *p* < 0.001) and weak for Aβ42/Aβ40 ratio (*ρ* = 0.39, *p* < 0.001). Plasma p-Tau181 and p-Tau181/Aβ42 concentrations were significantly increased while Aβ42/Aβ40 was significantly decreased (*p* < 0.001) in patients with AD dementia and prodromal AD, as well as in individuals with CSF abnormal amyloid concentrations (A +). Plasma p-Tau181 showed a robust performance in differentiating patients clinically diagnosed as AD (AUC = 0.89; 95% CI 0.83–0.94); A + vs. A − (AUC = 0.84, 95% CI 0.77–0.91) and also in predicting conversion to AD dementia in MCI patients (AUC = 0.89, 95% CI 0.81–0.96). When tested in the validation cohort, plasma p-Tau181 displayed 83.3% of the overall percentage of agreement according to amyloid status.

**Conclusions:**

Our results show that the measurement of p-Tau181 in plasma has great potential as a non-invasive prognostic screening tool for implementation in a clinical setting.

**Supplementary Information:**

The online version contains supplementary material available at 10.1186/s13195-024-01397-9.

## Background

Alzheimer’s disease (AD) is the most prevalent type of dementia, with aging as its principal risk factor [1]. Identifying individuals at an early stage of the disease, known as mild cognitive impairment (MCI), who will benefit from treatment, is particularly important for disease-modifying therapies that aim to slow disease progression [2, 3]. The neuropathological hallmark of AD consists of beta-amyloid (Aβ) plaques, intracellular aggregates of hyperphosphorylated tau protein (p-Tau) known as neurofibrillary tangles, and neurodegeneration. These pathological features can be indirectly assessed by quantifying these biomarkers in the cerebrospinal fluid (CSF) [1, 4]. Recent technological advancements have enabled the quantification of CSF-AD proteins, such as Aβ42, Aβ40, p-Tau, and total Tau [t-Tau], in the peripheral blood [5–7].

In the pursuit of good-performing blood-based markers, studies on plasma Aβ have yielded conflicting results, partly due to greater variations in Aβ isoforms in plasma compared to CSF [8–10]. While mass spectrometry-based Aβ assays have shown promise in predicting cognitive decline and PET amyloid positivity [11, 12], overall, plasma Aβ appears less robust and poses challenges for clinical implementation [13]. In regard to phosphorylated Tau, plasma p-Tau181 has shown potential as a prognostic tool, with baseline and longitudinal concentrations positively associated with AD dementia progression in MCI patients [14–16]. It also demonstrated effectiveness in discriminating patients in the AD continuum from other types of dementia [10, 17].

Notably, both plasma markers have provided valuable insights into amyloid abnormality, showing lower plasma Aβ concentrations and increased p-Tau181 in individuals with CSF and/or PET-positive [8, 9]. Plasma Aβ42/Aβ40 showcased good dynamic performance, with declining concentrations potentially proceeding brain amyloid accumulation by decades [15, 18]. Meanwhile, p-Tau181, especially when evaluated as p-Tau181/Aβ42, closely reflects current brain amyloid concentrations [15, 19].

Furthermore, the development of blood-based biomarkers for dementia diagnosis hinges on several key features: sensitivity for early detection, specificity for the disease, accessibility, and the ability to indicate disease progression [7, 20]. Current blood biomarker assays often use low-throughput and/or plate-based approaches designed for single-batch analysis to reduce variability, which can lead to limited scalability and longer turnaround times. Also, some prototype assays rely on proprietary or commercially unavailable reagents, further limiting their widespread use [21, 22]. However, the fully automated LUMIPULSE G platform, a widely used method for the quantification of CSF-AD biomarkers, offers high throughput, wide availability, and high reproducibility for blood-based biomarkers [22, 23]. With its convenient approach and adherence to standard laboratory techniques, this platform holds the potential to facilitate the implementation of blood tests for AD pathology prediction and diagnosis in clinical laboratories worldwide [6, 7].

In this study, we (1) evaluated, in an (retrospective) exploratory cohort (*n* = 138), the clinical performance of LUMIPULSE G plasma assays for amyloid and phosphorylated Tau as an indication of abnormal amyloid status, suggestive of brain AD pathology; (2) assessed the ability of these plasma markers to predict progression from MCI to AD dementia; (3) estimated the proportion of prevented CSF testing; and (4) subsequently confirmed these results in a (prospective) validation cohort attending the clinic for diagnostic evaluation (*n* = 72).

## Methods

### Subjects

Our sample consisted of 210 individuals recruited at the Neurology Department of Coimbra University Hospital. This sample was divided into two main cohorts: the exploratory cohort and the validation cohort.

The exploratory cohort consisted of 138 individuals (22 neurological controls [NC], 72 MCI, and 44 AD dementia subjects) who underwent a prospective follow-up and diagnosis supported by CSF-AD biomarkers. MCI was diagnosed in accordance with the clinical/neuropsychological framework for MCI within the syndromal categorical scheme, proposed by the NIA-AA criteria [2]. NC subjects were admitted due to acute or chronic headaches, and a lumbar puncture was performed as part of the routine diagnostic evaluation; their cytochemical evaluation was normal, and any major disease of the central nervous system was excluded. AD dementia subjects fulfilled the clinical diagnostic criteria for probable AD [24].

Patients diagnosed with MCI had biannual clinical observation and annual neuropsychological assessment to detect progression to AD dementia for at least 2 years. The cohort was divided into those individuals who were cognitively stable (*n* = 36) and those who developed AD dementia (*n* = 36), during an overall follow-up time of 6.4 ± 2.5 years. The conversion required fulfilling clinical diagnosis criteria for probable AD dementia [24] upon consensus among the clinicians and confirmed by the senior neurologist of our dementia clinic (IS) and was also described in a previous study [25].

In the validation cohort, 72 individuals with cognitive complaints were included and also investigated with CSF-AD biomarkers. This cohort reflects a broad diagnostic classification, encompassing individuals at various stages within the AD spectrum, including those potentially presenting co-pathologies.

At baseline, all individuals were stable, without acute comorbidities, and underwent a neurological evaluation performed by a behavioral neurologist, including detailed history from the patient and at least one other reliable source as well as clinical neurological examination, psychiatric evaluation, cognitive screening tests (such as the Mini-Mental State Examination [MMSE] and the Montreal Cognitive Assessment [MoCA]) and a comprehensive neuropsychological evaluation encompassing various domains of cognitive function [25].

The exclusion criteria consisted of any significant underlying medical or neurological illness shown through laboratory tests or imaging, major psychiatric disorders, and CT or MRI demonstration of significant vascular burden (history of stroke or extensive subcortical white matter lesions superior to 25% or by Fazekas scale ≥ 2) [26], and in the case of NC subjects, any cognitive disturbance.

The study was conducted according to the guidelines of the Declaration of Helsinki and approved by the Ethics Committee of Coimbra University Hospital (OBS.SF.228–2021).

### Laboratory determinations

#### CSF biomarkers determination

Samples were collected from patients and NC as part of their diagnostic examination between 2012 and 2021, and CSF concentrations of Aβ42, Aβ40, t-Tau, and p-Tau181 were determined in a routine setting. Standard pre-analytical and analytical procedures were followed according to the BIOMARKAPD guidelines for CSF-AD biomarkers [27]. Specifically, CSF samples were collected in sterile polypropylene tubes, immediately centrifuged at 1800* g* (10 min at 4 °C), aliquoted into polypropylene tubes, and stored at − 80 °C until analysis. CSF Aβ42, Aβ40, t-Tau, and p-Tau181 were measured separately by commercially available immunoassays (INNOTEST and LUMIPULSE, Fujirebio, Japan). External quality control of the assays was performed under the scope of the Alzheimer’s Association Quality Control Program for CSF Biomarkers [28].

CSF-AD biomarkers were determined using two different methods, and to be able to combine them, CSF samples originally quantified using the manual INNOTEST were re-assayed in the LUMIPULSE G600II platform at the same time as the plasma quantifications were performed. Method comparison estimation by Passing-Bablok regression has already been performed by Leitão et al. in 2019.

To classify CSF data, we employed the ATN scheme [3], which includes the Aβ42/Aβ40 ratio for assessing amyloid deposition (A), p-Tau181 for evidence of Tau aggregation (T), and t-Tau for neurodegeneration (N). Specific laboratory-established cutoffs for the LUMIPULSE platform [29, 30] were used to categorize marker concentrations as either normal ( −) or abnormal ( +). Abnormal markers were defined by concentrations below 0.068 for the Aβ42/Aβ40 ratio, above 51.2 for p-Tau181, and exceeding 354 for t-Tau.

#### Peripheral blood biomarker determination

Blood samples were collected into EDTA tubes on the same day as the lumbar puncture. These samples were centrifuged at 1800* g* (10 min at 4 °C), aliquoted into polypropylene tubes, and stored at − 80 °C until analysis.

Plasma concentrations of the analytes of interest were determined in the LUMIPULSE G600II platform (Fujirebio, Japan). For the exploratory study, the plasma samples were analyzed between October and November 2022, while for the validation cohort, the analysis took place between February and March 2023.

Plasma concentrations of p-Tau181, Aβ42, and Aβ40 were assessed simultaneously using the LUMIPULSE G pTau 181 plasma, LUMIPULSE G β-amyloid 1–40 plasma, and LUMIPULSE G β-amyloid 1–42 plasma research use only (RUO) assays, following the manufacturer’s instructions. Concentrations were determined via a lot-specific calibration curve, assayed in duplicate, and quality control procedures were performed at the beginning of each test day to ensure that control values (low and high) fitted the target ranges.

#### Statistical analysis

Statistical analysis was performed using the statistical software R (version 4.1.3). A two-tailed *p*-value less than 0.05 was considered statistically significant. To test for normal distribution, the Shapiro–Wilk test was used. As protein concentrations were not normally distributed, the groups were compared using the Wilcoxon rank-sum test with a Bonferroni correction and Kruskal–Wallis with Dunn tests. Relationships between the log-transformed protein values (except the ratios) were examined using Spearman’s rho (*ρ*). To determine the diagnostic ability of the markers, we developed receiver operating characteristics (ROC) curves and calculated the area under the curve (AUC). The comparison between curves was performed according to DeLong’s test for 2 correlated ROC curves, and a bootstrap procedure with 2000 permutations was applied. The overall percentage of agreement (OPA) was calculated as the sum of participants correctly classified by group over the total number of individuals. The estimation of the proportion of lumbar punctures saved using plasma biomarkers was derived from the ROC curves cutoffs with 95% sensitivity or 95% specificity. We calculated the number of samples with plasma marker concentrations below and above these cutoffs, to compute true negative + false negative (using the 95% sensitivity cutoff) and true positive + false positive (using the 95% specificity cutoff). For each proportion of saved exams, the “error rate” was calculated, based on those incorrectly classified (sum of the false negatives and the false positives).

## Results

### Population characteristics according to cognitive staging

The characteristics of the exploratory cohort (138 individuals: 22 NC, 72 MCI [36 converters to AD dementia and 36 non-converters], and 44 AD dementia) are displayed in Table 1. There were no significant differences between the groups according to basic demographics (age and sex) and time of follow-up in MCI patients. The concentrations of CSF-AD biomarkers (Aβ42/Aβ40 ratio, p-Tau181, and t-Tau) were in accordance with their clinical diagnosis (*p* < 0.001), with MCI-AD and AD dementia patients showing lower concentrations of Aβ42/Aβ40 ratio and higher concentrations of t-Tau and p-Tau18 in relation to both NC and MCI-St patients.

**Table 1:** Demographic and biomarker data of the exploratory cohort according to cognitive status and progression

Groups	NC (*n* = 22)	MCI-St (*n* = 36)	MCI-AD (*n* = 36)	AD (*n* = 44)	Test statistic (*p*-value)
*Demographic information*					
Age, years	63.0 (59.8–64.8)	64.5 (55.8–71.3)	68.5 (61.0–70.8)	64.0 (60.0–69.0)	0.131
Sex (% female)	55%	61%	72%	57%	0.456
Follow-up time, years	–	6.9 ± 1.9	5.9 ± 2.9	–	0.280
*CSF biomarker concentrations*					
CSF Aβ42/Aβ40 ratio	0.106 (0.097–0.114)	0.105 (0.077–0.112)	0.049 (0.038–0.057)	0.043 (0.038–0.051)	< 0.001
CSF t-Tau, pg/mL	165 (149–216)	303 (211–349)	520 (344–646)	719 (430–1010)	< 0.001
CSF p-Tau181, pg/mL	25.4 (23.0–32.7)	42.9 (31.8–53.8)	85.4 (55.3–116.8)	108.5 (72.8–168.5)	< 0.001
ATN classification: A-T-N-	100%	64%	6%	0%	< 0.001
A-T(^+^/_−_)N(^+^/_−_)	0%	17%	5%	0%	
A + T(^+^/_−_)N(^+^/_−_)	0%	19%	89%	100%	
*Blood-based biomarker concentrations*					
Plasma Aβ42/Aβ40 ratio	0.087 (0.083–0.092)	0.084 (0.079–0.094)	0.079 (0.074–0.083)	0.075 (0.070–0.083)	< 0.001
Plasma p-Tau181, pg/mL	1.4 (1.1–1.8)	1.5 (1.2–1.8)	2.8 (2.3–3.7)	3.3 (2.2–4.2)	< 0.001
Plasma p-Tau181/Aβ42 ratio	0.06 (0.04–0.10)	0.07 (0.06–0.11)	0.19 (0.13–0.37)	0.25 (0.17–0.40)	< 0.001

Data is presented as median (25th–75th percentiles) or as percentage. Tests used: Kruskal–Wallis (except follow-up time: Wilcoxon rank-sum) for continuous variables and Pearson’s chi-squared for nominal category

*Abbreviations*: *Aβ* amyloid beta, *AD* Alzheimer’s disease, *CSF* cerebrospinal fluid, *MCI-AD* mild cognitive impairment converters to AD dementia, *MCI-St* mild cognitive impairment stable, *NC* neurological controls, *p-Tau181* phosphorylated Tau protein in the position 181, *t-Tau* total Tau protein

**Fig. 1:**
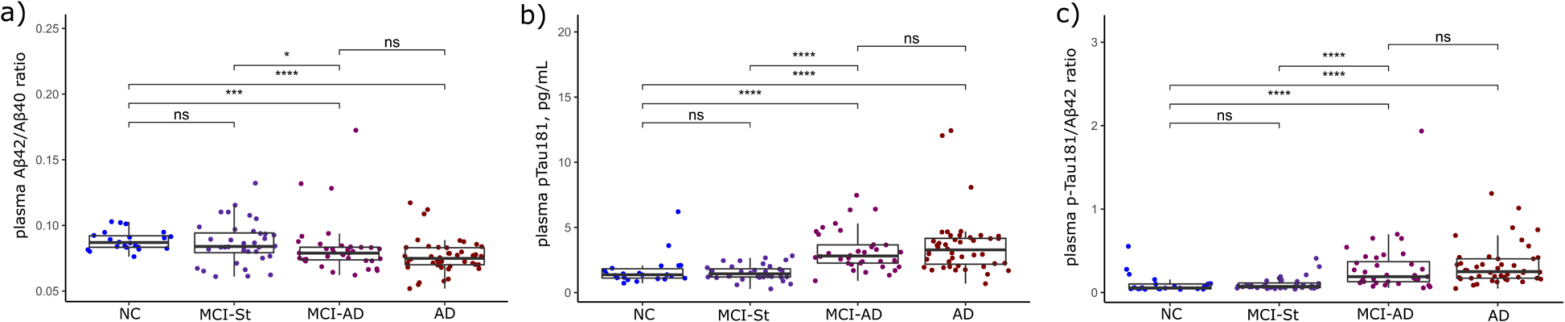
Plasma amyloid and phosphorylated Tau concentrations in the exploratory cohort according to cognitive status and progression. Concentrations depicted **A** Aβ42/Aβ40 ratio, **B** p-Tau181, and **C** p-Tau181/Aβ42 ratio. Data is presented in points as individual values and the spread of the distribution with quartiles and median by a boxplot categorized by NC (*n* = 22), MCI-St (*n* = 36), MCI-AD (*n* = 36), and AD dementia (*n* = 44). Group comparison was performed by Kruskal–Wallis test with a Bonferroni correction showing a *p*-value label of significance: *p < 0.05; **p < 0.01; ***p < 0.001; n.s., not significant. *Abbreviations*: Aβ, amyloid beta; AD, Alzheimer’s disease; MCI-AD, mild cognitive impairment that progressed to AD dementia; MCI-St, stable mild cognitive impairment; p-Tau181, phosphorylated tau protein in the position 181

Plasma p-Tau181 and p-Tau181/Aβ42 ratio displayed a similar behavior between the groups as for CSF (*p* < 0.001), with increased concentrations in MCI-AD and AD dementia patients in relation to both NC and MCI-St patients (also shown in Fig. 1). For plasma, Aβ42/Aβ40 ratio showed a decrease in MCI-AD vs. MCI-St patients and NC compared to AD patients. No differences were seen in the plasma markers between NC and MCI-St and also between MCI-AD and AD dementia patients.

When separating our sample set according to their amyloid status as normal (A − ; *n* = 58) and abnormal (A + ; *n* = 80), according to our validated cutoff for CSF Aβ42/Aβ40 ratio (Additional file 1: Table S1), consistent results for the plasma biomarkers were obtained. As seen in Fig. 1, a significantly decreased concentration of Aβ42/Aβ40 ratio was seen in A + patients, while p-Tau181 and the p-Tau181/Aβ42 ratio were significantly increased (*p* < 0.001).

### Relationship among AD core biomarkers

**Fig. 2:**
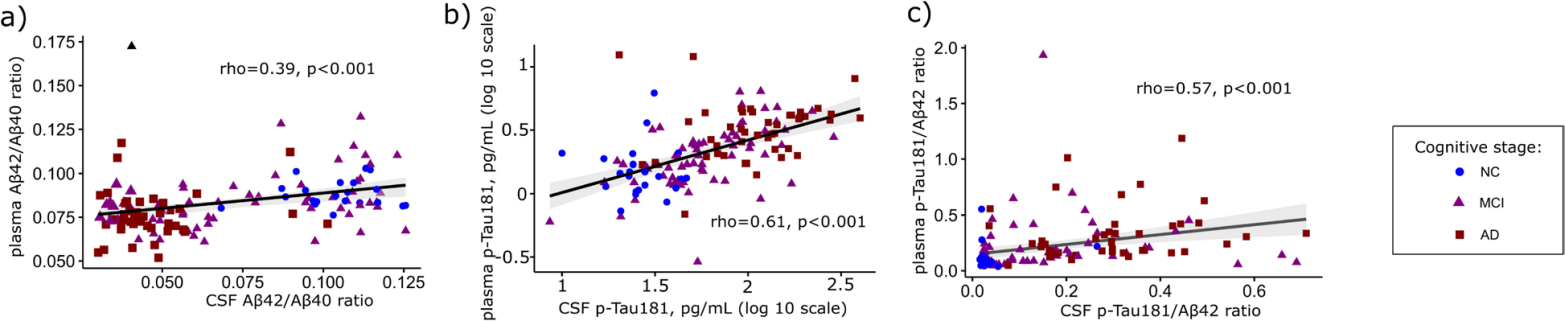
Associations between CSF amyloid and phosphorylated Tau with corresponding plasma concentrations in the exploratory cohort. **A** CSF and plasma Aβ42/Aβ40 ratio. **B** CSF and plasma p-Tau181 concentrations. **C** CSF and plasma p-Tau181/Aβ42 ratio. Graphs are presented with a logarithmic transformed axis, except for the ratios. Data displays individual values with mean regression and 95% prediction lines, with shapes corresponding to the cognitive stage (• NC, ▴ MCI, and ■ AD). Spearman correlation coefficients and *p*-values are presented for each graph. *Abbreviations*: Aβ, amyloid beta; AD, Alzheimer’s disease dementia; CSF, cerebrospinal fluid; MCI, mild cognitive impairment; NC, neurological control; p-Tau181, phosphorylated tau protein in the position 181

Figure 2 depicts the comparison of the different markers and ratios between fluids (CSF vs plasma), where p-Tau181 showed the highest correlation (*ρ* = 0.61, *p* < 0.001), followed by the p-Tau181/Aβ42 ratio with a moderate relation (*ρ* = 0.57, *p* < 0.001). Additionally, the Aβ42/Aβ40 ratio displayed a weak correlation (*ρ* = 0.39, *p* < 0.001).

Moreover, we also obtained significant (*p* < 0.001) moderate correlations between plasma p-Tau181 and the additional CSF-AD markers: Aβ42/Aβ40 ratio and t-Tau (*ρ* =  − 0.52 and *ρ* = 0.57, respectively; not shown).

### Diagnostic performance of plasma biomarkers

**Fig. 3:**
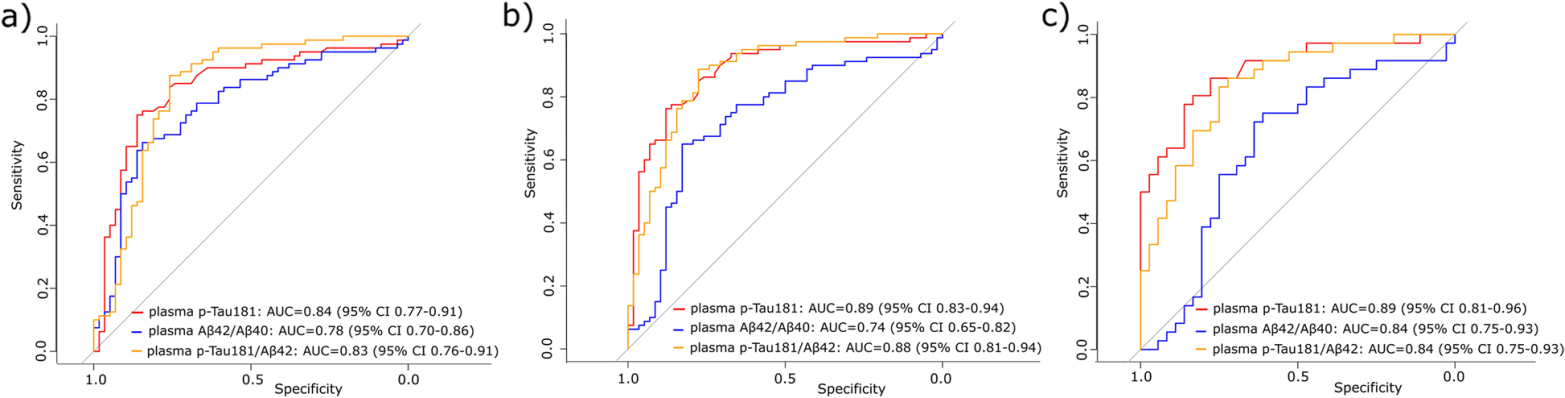
High diagnostic accuracy of plasma amyloid and phosphorylated Tau 181 in the exploratory cohort according to **A** amyloid status, **B** clinical condition, and **C** progression to AD dementia. Receiver operating curve (ROC) analyses presented with the area under the curve (AUC) with 95% confidence interval (CI) for plasma Aβ42/40 ratio, p-Tau181, and p-Tau181/Aβ42 ratio. Amyloid status was determined according to reference values of CSF Aβ42/Aβ40 ratio for dichotomization into negative (*n* = 58) and positive (*n* = 80). Clinical condition was determined according to their probable clinical diagnosis, grouped as NC + MCI-Stable (*n* = 58) vs. MCI-AD + AD (*n* = 80). MCI progression was determined, dividing patients into those who remained cognitively stable (MCI-Stable; *n* = 36) and those that developed AD-dementia (MCI-AD; *n* = 36). *Abbreviations*: Aβ, amyloid beta; AD, Alzheimer’s disease; CSF, cerebrospinal fluid; MCI, mild cognitive impairments; p-Tau181, phosphorylated tau protein in the position 181

When testing the accuracy of plasma biomarkers (Aβ42/Aβ40, p-Tau181/Aβ42, and p-Tau181) in predicting amyloid status defined by the CSF Aβ42/Aβ40 ratio, AUC values were 0.78 (95% confidence interval [CI] 0.70–0.86) for plasma Aβ42/Aβ40, 0.83 (95% CI 0.76–0.91) for plasma p-Tau181/Aβ42, and 0.84 (95% CI 0.77–0.91) for plasma p-Tau181, as seen in Fig. 3A.

As no differences were found in plasma biomarker concentrations between NC and MCI-St and between MCI-AD and AD patients, we assessed the diagnostic accuracy of the plasma biomarkers based on these two categories: NC + MCI-St (*n* = 58) vs. AD + MCI-AD (*n* = 80). As shown in Fig. 3B, AUC values were 0.74 (95% CI 0.65–0.82) for plasma Aβ42/Aβ40, 0.88 (95% CI 0.81–0.94) for plasma p-Tau181/Aβ42, and 0.89 (95% CI 0.83*–*0.94) for plasma p-Tau181.

Regarding MCI patients, Fig. 3C displays the discrimination between converters (*n* = 36) and non-converters (*n* = 36) with similar AUC values as before, 0.84 (95% CI 0.75–0.93) for plasma Aβ42/Aβ40, 0.84 (95% CI 0.75–0.93) for plasma p-Tau181/Aβ42, and 0.89 (95% CI 0.81–0.96) for plasma p-Tau181.

Overall, plasma p-Tau181 displayed the best performance distinguishing between positive and negative classes in the three conditions studied (amyloid status, clinical condition, and progression to dementia). Additionally, when comparing the curves from plasma p-Tau181 with the ratio p-Tau181/Aβ42, no significant differences were obtained (*p* = 0.70, 0.92, and 0.33, respectively).

As done above to predict amyloid status based on CSF Aβ42/Aβ40 ratio, we also performed a ROC analysis to test the accuracy of the plasma biomarkers in predicting tauopathy based on CSF p-Tau181 concentrations and neurodegeneration based on CSF t-Tau (curves displayed in Supplementary materials) and calculated plasma cutoffs with 95% sensitivity and 95% specificity for each. We then estimated the proportion of potentially saved CSF tests based on the number of samples with plasma biomarker concentrations below and above these cutoffs, as well as their error rate (ER). The estimated proportion of saved CSF Aβ42/Aβ40 ratio was 37% using plasma p-Tau181/Aβ42 ratio (ER, 14%) and 38% with plasma p-Tau181 (ER, 13%), whereas plasma Aβ42/Aβ40 ratio presented an error rate higher to the calculated proportion (15%; ER, 28%). The estimated proportion of saved CSF p-Tau181 quantification was 38% using plasma p-Tau181 (ER, 13%) and 35% using plasma p-Tau181/Aβ42 ratio (ER, 13%). For neurodegeneration, plasma p-Tau181 displayed a saved proportion of 19% (ER, 31%) and plasma p-Tau181/Aβ42 of 30% (ER, 17%).

### Classification agreement in a validation cohort using plasma phosphorylated Tau

In the validation cohort, we included 72 individuals with cognitive complaints, dichotomized according to their amyloid status (41 A − vs. 31 A +). The characteristics of this cohort are shown in Additional file 1: Table S1.

Due to the robust results obtained with plasma p-Tau181 in the exploratory cohort, we decided to focus solely on this protein for the validation study. Therefore, the percentage of agreement according to the diagnostic ability reported for amyloid status (AUC = 0.84, 95% CI 0.77–0.91) was 80% for A − and 87% for A + , with an OPA of 83.3%.

Additionally, using the 95% sensitivity and 95% specificity cutoffs, plasma p-Tau181 showed an estimated proportion of saved traditional exams for amyloid status of 40% (ER, 17%) and 43% (ER, 10%) for tauopathy.

## Discussion

The quantification of CSF proteins associated with AD’s neuropathological changes is a routine practice in many healthcare institutions [3]. Extensive evidence supports their high accuracy in aiding clinical diagnosis, frequently in combination with neuropsychological measures and/or cerebral imaging [2, 3]. With increasing demand and the need for standardization, fully automated assays brought necessary advantages to CSF-AD biomarkers quantification [30]. However, the invasiveness of lumbar punctures underlines the need for more sensitive, standardized, and high-throughput methods for the transition from CSF to blood-based markers of AD pathology.

Our results confirm the clinical potential of assessing plasma Aβ42, Aβ40, and p-Tau181 concentrations using the fully automated LUMIPULSE G600II platform. In our retrospective study (*n* = 138), we observed decreased amyloid (Aβ42/Aβ40) and increased p-Tau181 concentrations in patients within the AD spectrum (both AD dementia and prodromal stages), as well as in patients with evidence of brain amyloid pathology. We found strong associations between plasma p-Tau181 and CSF p-Tau181 concentrations, while correlations for Aβ peptides in both fluids were weaker. The diagnostic ability of plasma p-Tau181 alone or in combination with plasma Aβ42 was optimal to predict amyloid status, assessed through CSF Aβ42/Aβ40, clinical AD diagnosis, and also conversion to AD dementia in MCI patients. Finally, the potential of plasma p-Tau181 to detect brain amyloid pathology was validated in an independent cohort, with consistent results showing an overall percentage of agreement of 83.3%.

The use of LUMIPULSE plasma AD biomarkers represents a novel approach, and although limited studies have been published, recent results [23, 31] emphasized the favorable results over standardization of sample collection and storage, which further validates the robustness of the assays and the methodology employed in our work. Also, our findings align with those of Wilson and collaborators (2022). They specifically focused on plasma p-Tau181 concentrations and observed gradual increases along the AD continuum, identified moderate positive associations between CSF and plasma, and reported high accuracy in distinguishing AD-related changes, which closely reflect amyloid abnormality.

Another fully automated plasma biomarker, the Elecsys prototype immunoassay panel has been described [32], demonstrating high accuracy in identifying amyloid positivity. However, in contrast to our findings, the best discriminator for A + versus A − participants in that prototype was Aβ42/Aβ40. Additionally, while the combination of markers (p-Tau181, p-Tau217, and Aβ42/Aβ40) proved optimal for predicting AD dementia in MCI patients in their study, our research suggests that p-Tau181 alone provides the best predictive value. This lack of improved clinical performance by the combination of markers may be attributed to their redundancy as diagnostic indicators, potentially capturing similar aspects of AD pathology. Additionally, the characteristics of our sample population may favor p-Tau181 over the combination, as several factors such as disease progression, heterogeneous patient profiles, or different stages of AD pathology can influence the relative performance of each marker [4].

Despite platform differences, fully automated blood-based biomarker assays designed for routine clinical practice can achieve comparable accuracy in detecting amyloid brain pathology and predicting future AD dementia. Previous studies on research-targeted platforms, including single molecule array (Simoa), reported AUCs ranging from 0.70 to 0.95 in plasma marker combinations [10, 21, 33]. Similarly, immunoassay-based platforms (Elecsys) exhibited AUCs exceeding 0.70 [13, 32], while mass-spectrometry-based assays showed AUCs above 0.80, specifically for plasma amyloid [12, 13]. In our study, plasma p-Tau181 (alone and in combination with Aβ42) demonstrated comparable accuracy for amyloid and tau status, with AUCs of 0.84 and 0.83, respectively. However, it underperformed as a measure of neurodegeneration. This shortfall can be attributed to the broad nature of neuronal loss, unspecific to AD [3, 9].

With the intention of replicating the results from Altomare and collaborators (2023), we also showed the potential of plasma p-Tau181 for preventing the quantification of CSF amyloid and phospho tau, in 38% of our exploratory cohort and 40–43% of our validation sample. While the concept of “lumbar punctures saved using plasma biomarkers” can provide potential cost savings or resource allocation, it is important to acknowledge that the metric is influenced by the prevalence of the disease within the population being tested. Additionally, it is necessary to be cautious in interpreting these results due to the small sample size, and the considerable error rates observed, it is noticeable that plasma p-Tau181 exhibited lower error rates in comparison with the amyloid markers. In contrast, the amyloid markers have the potential to convey misclassifications due to their narrow dynamic range [13]. Therefore, the combination of several diagnostic tools and comprehensive clinical assessments may be necessary to achieve a more accurate diagnosis.

Moreover, we performed a small validation of our cutoffs in an independent cohort, focused on plasma p-Tau181, which showed strong accuracy by correctly classifying amyloid pathology in 83.3% of the sample. This high percentage of agreement, with the diagnostic ability previously reported, provides further support for the use of plasma p-Tau181 as a reliable diagnostic tool. Moreover, the estimated proportion of potential saved CSF quantifications (for both amyloid status and tauopathy) could possibly reduce the need for a lumbar puncture in those selected individuals. Overall, these results highlight the promise of plasma p-Tau181 as a non-invasive biomarker for early diagnosis and monitoring along the AD continuum. Despite the high precision of the LUMIPULSE G platform, as evidenced by its low intra-and inter-instrument coefficients of variability, caution should be exercised when applying a predefined cutoff and measuring samples in daily or weekly batches over an extended period. While the results of our validation cohort (OPA = 83.3%), which used a different batch of reagents compared to the exploratory cohort, are promising, additional studies are necessary to examine the accuracy and robustness of the assay when analyzing samples over longer periods of time.

The outcomes of our study align with the existing literature [15, 16, 21, 34, 35], where p-Tau is gathering sufficient evidence suggestive of its potential for implementation in a clinical setting, as a screening tool specific for AD. Even though p-Tau217 has shown better performance in the prediction of future cognitive decline in pre-symptomatic and early stages [18, 34], p-Tau181 provides advantages from its cohesiveness from its comparability with traditional gold standard biomarkers, such as CSF p-Tau181 quantification and other modalities (i.e., Tau-PET imaging), and the possibility of performing confirmatory studies by post-mortem neuropathology. Additionally, the automation of protein quantification, as represented by the use of the LUMIPULSE G platform, simplifies lab methodologies, supports standard operating procedures, promotes accessibility, and gives more precise results.

It is important to consider the confounding effects impacting blood-based AD core biomarkers. In the case of p-Tau181, higher concentrations have been seen in patients with chronic kidney disease, which affects the clearance of proteins [36, 37], and in those with higher body mass index, resulting in lower concentrations [33, 36]. When establishing clinical predictive models, both these variables may need to be factored in, as well as the usual co-variates of CSF studies related to AD dementia (age, sex, formal years of education, and apolipoprotein ε4). However, recent evidence does not indicate a significant improvement in the prediction of AD in accounting for those plasma-related variables, suggesting a minimal influence [33].

Our work presents several limitations. First, by being a single-center study, our sample size was relatively small, which may impair the generalizability of our results. Second, we were unable to evaluate the effects of comorbidities and risk factors associated with blood-based biomarkers (i.e., body mass index) due to inaccessibility to pertinent information. Third, since this is an exploratory study with a convenience sample, it requires further confirmatory testing with larger and more diverse samples, including other types of dementia.

The screening strategy suggested for blood biomarkers implementation in memory clinics is expected to follow a systematic approach, where the identification of patients with uncertain and abnormal concentrations of these markers would be indicative of potential risk. Subsequentially, they would undergo confirmatory testing using CSF or PET imaging [9, 38, 39]. In our study, we established thresholds carefully aimed at maximizing diagnostic accuracy. Through a validation assessment, we ensured the generalizability and robustness of these values within our population. In the future, it will involve iterative analyses to refine and optimize these measures, guaranteeing their effectiveness and applicability in clinical practice.

## Conclusions

Plasma p-Tau181 as measured with LUMIPULSE G (alone or in combination) presented robust evidence to be implemented as a screening tool for diagnosis and prediction of brain AD pathology, allowing for a more frequent sampling, due to the easier and less invasive collection, and facilitating wide accessibility to biomarker testing.

### Supplementary Information

**Additional file 1: Table S1.** Demographic and biomarker data of the exploratory and validation cohorts according to amyloid status. **Fig. S1.** Plasma amyloid and phosphorylated Tau 181 levels in the exploratory cohort according to amyloid status. Concentrations depicted: A) Aβ42/Aβ40 ratio, B) p-Tau181, and C) p-Tau181/Aβ42 ratio. Data is presented in points as individual values and the spread of the distribution with quartiles and median by a boxplot categorized by CSF Aβ42/Aβ40 dichotomization as amyloid status negative (*n*=58) and positive (*n*=80). Group comparison was performed by Wilcoxon rank-sum test with a Bonferroni correction showing a p-value label of significance: * p<0.05; ** *p*<0.01; *** *p*<0.001; n.s. as not significant. Abbreviations: Aβ = amyloid beta; CSF = cerebrospinal fluid; p-Tau181 = phosphorylated tau protein in the position 181. **Fig. S2.** Moderate diagnostic accuracy of plasma amyloid and phosphorylated Tau 181 in the exploratory cohort according to: A) tauopathy, and B) neurodegeneration. Receiver-operating characteristics (ROC) curve analyses presented with the area under the curve (AUC) with 95% confidence interval (CI) for plasma p-Tau181 and p-Tau181/Aβ42 ratio. Tauopathy was determined according to reference values of CSF p-Tau181 for dichotomization into negative (*n*=59) and positive (*n*=79). Neurodegeneration was determined according to reference values of CSF t-Tau for dichotomization into negative (*n*=64) and positive (*n*=74). Abbreviations: Aβ = amyloid beta; CSF = cerebrospinal fluid; p-Tau181 = phosphorylated tau protein in the position 181; t-Tau = total Tau protein.

## Data Availability

The dataset used and/or analyzed during the current study is available from the corresponding author upon reasonable request.

## Abbreviations

A: Evidence of amyloid deposition under the category ATN
Aβ: Amyloid beta protein
AD: Alzheimer’s disease
AUC: Area under the curve
CI: Confidence interval
CSF: Cerebrospinal fluid
CT: Computerized tomography
ER: Error rate
MCI: Mild cognitive impairment
MCI-AD: Mild cognitive impairment patients that converted to Alzheimer’s disease dementia
MCI-St: Non-converters mild cognitive impairment patients
MMSE: Mini-Mental State Examination
MoCA: Montreal Cognitive Assessment
MRI: Magnetic resonance imaging
N: Evidence of neurodegeneration under the category ATN
NC: Neurological controls
OPA: Overall percentage of agreement
PET: Positron emission tomography
p-Tau181: Phosphorylated Tau protein at threonine-181
ROC: Receiver operating characteristic
RUO: Research use only
T: Evidence of Tau aggregation under the category ATN
t-Tau: Total Tau protein
