# Supplementary material for: Exploring the potential of fully automated LUMIPULSE G plasma assays for detecting Alzheimer’s disease pathology
Source: Alzheimers Res Ther. 2024 Mar 7;16:51. doi: 10.1186/s13195-024-01397-9 (PMC10918996; doi:10.1186/s13195-024-01397-9)
Supplement: Supplementary file 1 — Additional file 1: Table S1. Demographic and biomarker data of the exploratory and validation cohorts according to amyloid status. Fig. S1. Plasma amyloid and phosphorylated Tau 181 levels in the exploratory cohort according to amyloid status. Concentrations depicted: A) Aβ42/Aβ40 ratio, B) p-Tau181, and C) p-Tau181/Aβ42 ratio. Data is presented in points as individual values and the spread of the distribution with quartiles and median by a boxplot categorized by CSF Aβ42/Aβ40 dichotomization as amyloid status negative (n=58) and positive (n=80). Group comparison was performed by Wilcoxon rank-sum test with a Bonferroni correction showing a p-value label of significance: * p<0.05; ** p<0.01; *** p<0.001; n.s. as not significant. Abbreviations: Aβ = amyloid beta; CSF = cerebrospinal fluid; p-Tau181 = phosphorylated tau protein in the position 181. Fig. S2. Moderate diagnostic accuracy of plasma amyloid and phosphorylated Tau 181 in the exploratory cohort according to: A) tauopathy, and B) neurodegeneration. Receiver-operating characteristics (ROC) curve analyses presented with the area under the curve (AUC) with 95% confidence interval (CI) for plasma p-Tau181 and p-Tau181/Aβ42 ratio. Tauopathy was determined according to reference values of CSF p-Tau181 for dichotomization into negative (n=59) and positive (n=79). Neurodegeneration was determined according to reference values of CSF t-Tau for dichotomization into negative (n=64) and positive (n=74). Abbreviations: Aβ = amyloid beta; CSF = cerebrospinal fluid; p-Tau181 = phosphorylated tau protein in the position 181; t-Tau = total Tau protein. [file 13195_2024_1397_MOESM1_ESM.pdf]

Supplementary Material

Supplementary Table: Demographic and biomarker data of the exploratory and validation cohorts according to amyloid status.

Supplementary Figure 1: Plasma amyloid and phosphorylated Tau 181 levels in the exploratory cohort according to amyloid status.

Supplementary Figure 2: Moderate diagnostic accuracy of plasma amyloid and phosphorylated Tau 181 in the exploratory cohort according to: A) tauopathy, and B) neurodegeneration.

Supplementary Table – Demographic and biomarker data of the exploratory and validation cohorts according to amyloid status.

	EXPLORATORY COHORT			VALIDATION COHORT		
**Groups**	**A- (n=58)**	**A+ (n=80)**	**Test statistic (p-value)**	**A- (n=41)**	**A+ (n=31)**	**Test statistic (p-value)**
Demographic information						
Age, years	63.5 (61.0-68.8)	65.0 (60.0-70.0)	0.528	62.0 (55.7-65.8)	71.0 (65.5-75.0)	<0.001
Sex (% female)	64%	60%	0.434	71%	68%	0.785
CSF ATN classification						
T-N-	86%	9%	<0.001	88%	10%	<0.001
T-N+	2%	1%		0%	6%	
T+N-	2%	8%		0%	6%	
T+N+	10%	82%		12%	78%	
Blood-based biomarker concentrations						
plasma Aβ42/Aβ40 ratio	0.09 (0.08-0.09)	0.08 (0.07-0.08)	<0.001	-	-	-
plasma p-Tau181, pg/mL	1.49 (1.18-1.91)	2.92 (2.21-4.08)	<0.001	1.15 (0.86-1.50)	2.22 (1.96-2.56)	<0.001
plasma p-Tau181/Aβ42 ratio	0.07 (0.05-0.11)	0.20 (0.15-0.34)	<0.001	-	-	-

Data is presented as median (25th-75th percentiles) or percentage. Dichotomized amyloid status was obtained following reference values of CSF Aβ42/Aβ40 for the population of our center (published study by Baldeiras et al. 2018). Tests used: Wilcoxon rank-sum for continuous variables and Pearson's chi-squared for nominal category. Abbreviations: Aβ = amyloid beta; A = amyloid status; CSF = cerebrospinal fluid; p-Tau181 = phosphorylated Tau protein in the position 181; t-Tau = total Tau protein.

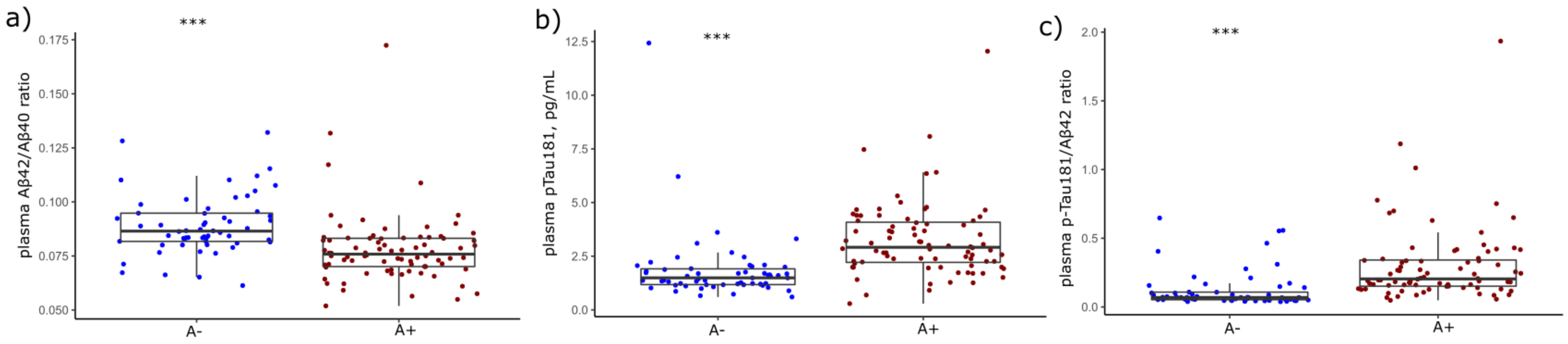

Supplementary Figure 1: Plasma amyloid and phosphorylated Tau 181 levels in the exploratory cohort according to amyloid status. Concentrations depicted: A) Aβ42/Aβ40 ratio, B) p-Tau181, and C) p-Tau181/Aβ42 ratio. Data is presented in points as individual values and the spread of the distribution with quartiles and median by a boxplot categorized by CSF Aβ42/Aβ40 dichotomization as amyloid status negative (n=58) and positive (n=80). Group comparison was performed by Wilcoxon rank-sum test with a Bonferroni correction showing a p-value label of significance: * p<0.05; ** p<0.01; *** p<0.001; n.s. as not significant. Abbreviations: Aβ = amyloid beta; CSF = cerebrospinal fluid; p-Tau181 = phosphorylated tau protein in the position 181.

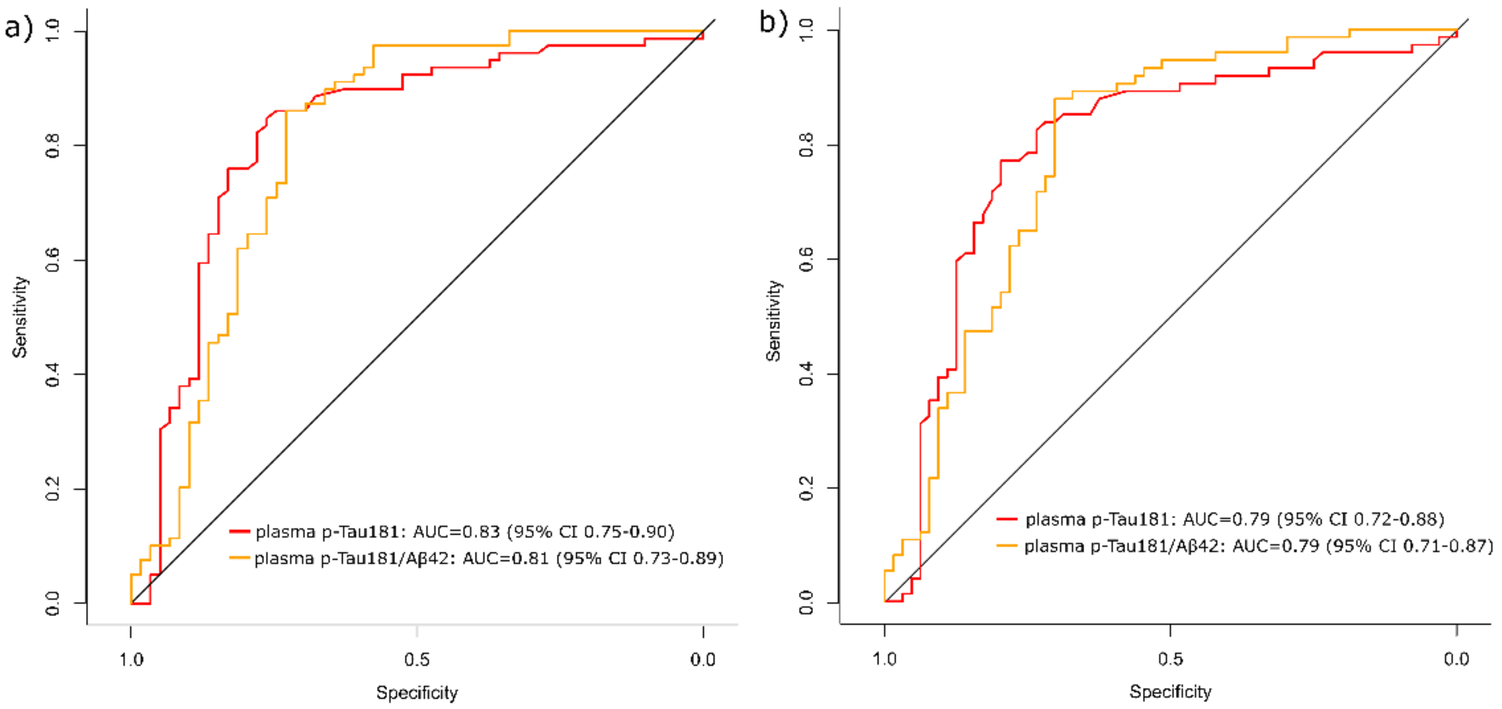

Supplementary Figure 2: Moderate diagnostic accuracy of plasma amyloid and phosphorylated Tau 181 in the exploratory cohort according to: A) tauopathy, and B) neurodegeneration. Receiver-operating characteristics (ROC) curve analyses presented with the area under the curve (AUC) with 95% confidence interval (CI) for plasma p-Tau181 and p-Tau181/Aβ42 ratio. Tauopathy was determined according to reference values of CSF p-Tau181 for dichotomization into negative (n=59) and positive (n=79). Neurodegeneration was determined according to reference values of CSF t-Tau for dichotomization into negative (n=64) and positive (n=74). Abbreviations: Aβ = amyloid beta; CSF = cerebrospinal fluid; p-Tau181 = phosphorylated tau protein in the position 181; t-Tau = total Tau protein.
